# A qualitative study of carers’ experiences of dementia cafés: a place to feel supported and be yourself

**DOI:** 10.1186/s12877-017-0559-4

**Published:** 2017-07-25

**Authors:** Nan Greenwood, Raymond Smith, Farrukh Akhtar, Angela Richardson

**Affiliations:** Faculty of Health, Social Care and Education, Kingston University and St George’s, University of London, 6th Floor Hunter Wing, St George’s, University of London, Cranmer Terrace, London, SW17 0RE UK

**Keywords:** Carers, Caregivers, Dementia cafés, Alzheimer’s cafés, Experience, Qualitative

## Abstract

**Background:**

Unpaid, informal carers or caregivers play an important role in supporting people living with dementia but the role can be challenging and carers themselves may benefit from support. Alzheimer’s, dementia or memory cafés are one such form of support . These cafés are usually provided in the voluntary sector and are a place where people with dementia and those supporting them, usually family carers, can meet with others in similar situations.

**Methods:**

Using semi-structured interviews, this qualitative study explored the experiences of 11 carers from five dementia cafés in and around London, England.

**Results:**

Thematic analysis resulted in the identification of four key themes. Cafés provide a relaxed, welcoming atmosphere where carers can go where they feel supported and accepted. Café attendance often brought a sense of normality to these carers’ lives. Carers and those they care for look forward to going and often enjoy both the activities provided and socialising with others. Other highlighted benefits included peer support from other carers, information provision and support from the volunteer café coordinators. Despite diversity in how the cafés were run and in the activities offered, there were many reported similarities amongst carers in the value ascribed to attending the cafés.

**Conclusions:**

Dementia cafés appear to be a valuable, perhaps unique form of support for carers giving them brief respite from their caring role. Future research incorporating mixed methods is needed to understand the perspectives of those living with dementia.

**Electronic supplementary material:**

The online version of this article (doi:10.1186/s12877-017-0559-4) contains supplementary material, which is available to authorized users.

## Background

With ageing populations, increasing numbers of people are living with dementia [1]. Much of the support for people with dementia living at home is provided by informal carers, usually families and friends [2]. Caring is often recognised as challenging [3] but increasingly the rewards of caring are also being highlighted [4, 5].

Being a carer for someone living with dementia can be more demanding than other caring relationships, with negative outcomes correlated with cognitive impairment and challenging behaviour of the person living with dementia [6]. These negative impacts are well documented. These include reductions in quality of life and emotional health [7], physical health [8] and negative financial consequences [9] with an increased risk of developing dementia themselves [10]. Social isolation [11] can be particularly important as carers often may not want to leave the person they care for and can be reluctant to go out because of changes in their behaviour [12]. Furthermore, people living with dementia and their carers report both experiencing stigma once diagnosed [13] and reduced social support [11]. Despite this, increasingly, with reductions in services, carers are expected to rely on their social networks for support [14].

Recognition of the negative consequences of caring has led to the development of supportive psychosocial interventions both specifically for carers and for the person living with dementia and carers together [15]. However, the evidence for their effectiveness is mixed. A comprehensive review of both randomised and non-randomised controlled trials suggested that, overall, individually tailored interventions lead to better outcomes. Additionally, psychoeducational interventions were beneficial for carers and can delay institutionalisation of the person living with dementia [3]. Similarly, a recent systematic review of reviews [16] found that well-structured, multi-component interventions can also delay institutionalisation and also help to maintain carers’ emotional health. The authors also suggest that interventions provided as part of a support group can increase their effectiveness in, for example, improving carer psychological health [16].

Alzheimer’s, dementia or memory cafés are one example of supportive intervention both for people with dementia and their carers. They were first developed approximately 20 years ago in the Netherlands [17]. The first café in the United Kingdom (UK) was established in 2000 and since then many more have been set up [18]. Most cafés in the UK are provided by the voluntary sector and have a mixture of volunteer and paid staff who are variously known as managers, café organisers and volunteer coordinators. These staff organise activities, greet attendees, provide refreshments and generally offer support. The activities available vary but can include music, singing, quizzes, gardening and information provision. Cafés are usually subsidised but may ask attendees for a relatively small contribution towards their running costs. Frequency of meetings is variable but they typically meet once or twice a month. The attendees can suggest activities they would like to see at the cafés and some talk directly to the cafés coordinators about their needs. The cafés are often held in community halls or libraries making them relatively easy for people living with dementia and their carers to access.

Some cafés have diverged from the original model in terms of café frequency and types of activities offered but the basic premise remains the same – gatherings for people living with dementia and their carer which *‘are held in a friendly, social, café-like atmosphere, where people can converse, listen to themed talks or interviews, enjoy refreshments and music, and come and go freely.*’ [18:443].

Intuitively dementia cafés have a lot of appeal but there is little research investigating the overall experiences – both benefits and downsides - of carers of people living with dementia attending them. Advocates tend to espouse the value of cafés but do not always offer the evidence to support their claims [18]. An article intended for those wanting to develop dementia cafés and which describes a café set up in London, England [19] suggests that dementia cafés may be more acceptable than day centres for people who have recently been diagnosed with dementia making them particularly suitable as an entry point to other services. The authors also emphasised the diversity of models with some carers using cafés more as a form of respite whilst others value shared activities. More recently, using mixed methods, an evaluation of three cafés in Australia using focus groups and surveys reported that they increased social inclusion and peer support, prevented isolation and enhanced attendees’ emotional wellbeing. They also demonstrated that the cafés improved attendees’ understanding and knowledge about dementia and facilitated access to other services. However, cafés did not meet everyone’s requirements particularly in relation to cultural and linguistic needs [20].

In summary, the available evidence suggests that dementia cafés can benefit carers of people living with dementia but their overall experiences using an unstructured approach and from carers’ perspectives have not been explored in-depth. Using semi-structured interviews, the aims of this study were therefore to investigate in-depth informal carers’ experiences of attending cafés.

## Methods

### Participant recruitment

There are approximately 70 dementia cafés in greater London hosted in a range of locations, with varying activities and meeting frequency. Some are primarily funded by local authorities with support from third sector organisations. The cafés we recruited from were similarly variable with some suggesting attendees pay notional amounts for refreshments. The café programmes also varied in the amount of structure with some placing greater emphasis on activities and others on social support. Potential dementia cafés where carers might be interviewed were identified through a combination of internet searches and ‘snowballing’. We used convenience sampling - the paucity of research in the area means we had no useful evidence of café characteristics that would make purposive sampling appropriate. Identified café managers were contacted to ask if they would allow us to recruit carers from their café by a researcher attending a meeting at the café. Where permission was granted, a researcher attended the café and provided potential carer participants with detailed, written information about the study and answered any questions. Carers were not given any incentive for participating. They were given at least 24 h to read the information and to decide whether they wished to take part in a single interview. Written consent was always given.

### Inclusion criteria

To be included in the study, participants had to be informal carers of someone living with dementia, able to speak English and had to have attended a dementia café at least three times over 6 months. This time was considered sufficient for carers to have gained an impression of the café and to have attended a variety of meetings with a range of activities.

### The interviews

We selected semi-structured interviews to encourage carers to speak in depth whilst ensuring they could bring up aspects of their experiences not included in the topic guide.

The topic guide was designed specifically for the study (See Additional file 1) and developed around the research aims and the available literature. Using the topic guide facilitated consistency between the interviewers and ensured that topics previously identified as important were covered [21]. Carers were asked open-ended questions for example: ‘Can you tell me why you first decided to come to the dementia café?’; ‘How have you found attending the café?’ and ‘What do you enjoy most about attending?’ At the end of the interview, they were also asked if there was anything else they wanted to add about their experiences.

The digitally recorded semi-structured in-depth interviews were carried out by three experienced interviewers all of whom were familiar with working with informal carers either as researchers, clinically or working in social care.

### Data analysis

All interviews were transcribed and anonymised. Analysis was thematic [22] and started during data collection to ensure that the topic guide reflected issues relating to the cafés that were of importance to carers. The research team read the transcripts to ensure familiarisation with the data. The transcripts were then shared out ensuring that all were analysed independently by a minimum of three researchers. Initial codes such as ‘benefits of attending’ and ‘trust’ were then generated by each researcher in an iterative process whereby the transcripts were repeatedly revisited to identify themes and conceptual relationships. The initial themes were reviewed against the data by two members of the team and revised where needed. This was an iterative process and involved the researchers checking that the themes fitted together meaningfully, that there were clear distinctions between themes and that the same data was not appearing in multiple themes. Key themes were then defined and named and the internal consistency of the themes was then checked finally against the data [22]. The entire team discussed these final themes and after clarification, consensus about the salience of the themes was achieved.

To enhance the analysis and help understand the identified themes, there was an active attempt to identify any negative, ‘outlier’, or ‘deviant’ participants in terms of their experiences of the cafés [23].

It was striking that although three researchers undertook the interviews with carers who attended cafés operating in a variety of ways, there was considerable similarity in the themes identified. This suggests that we have highlighted the essential, central aspects of carers’ experiences with dementia cafés. Further, the researchers came from a range of disciplines, which helped foster diversity in perspectives, encouraging debate and enhancing researcher reflexivity [24].

Ethical approval for the study was given by the Faculty of Health, Social Care and Education Research Ethics Committee, Kingston University and St George’s University of London. All interviews were undertaken with written informed consent. Confidentiality and anonymity were assured and the fact that the participants could terminate the interview at any time without providing an explanation was highlighted.

## Results

Carers were recruited from five cafés in and around London, England. Ten participants were interviewed in their own homes and one at a day centre. Interviews were variable in length (24–62 min) lasting an average of 42 min.

### The carer participants

All 11 carers who expressed an interest in participating were interviewed. All but one of the carers were family members – mostly spouses or partners and adult children. Eight were female and three were male. All were aged over 40 and most were aged between 61 and 80 years. When asked to describe their ethnicity, carers responded with a mixture of ethnic groups and nationalities. Most described themselves as ‘White’ (White British, White English and White Irish). For all but one, English was their first language. All participants had been caring for two or more years with most caring for over 4 years. Carers’ demographic details are available in Table 1.

**Table 1:** Carer participant demographic details

*n* = 11	Carers (%)
Age in years (categorised)	
41–50	2 (18.2)
51–60	2 (18.2)
61–70	3 (27.3)
71–80	4 (36.3)
Gender	
Female	8 (72.7)
Male	3 (27.3)
Ethnicity	
White British	8 (72.7)
Black British	2 (18.2)
South Asian	1 (9.1)
First language	
English	10 (90.9)
Bengali	1 (9.1)
Relationship to the person living with dementia	
Spouse	5 (45.45)
Adult child	5 (45.45)
Friend	1 (9.1)
Length of time caring (years)	
1–5	7 (63.6)
6–10	3 (27.3)
11+	1 (9.1)

### Themes

Four main themes were identified: An opportunity for enjoying themselves and to switch off from being a carer; cafés as normalising living with dementia; peer support; developing social networks and reducing social isolation. These are described below with anonymised quotes using pseudonyms for all names whether for participants or other people they were referring to.

### Carers’ lives

Before describing the themes, it is helpful to consider them in the context of carers’ lives. Carers situations and support needs varied. Most of the carers were retired or not in paid employment. One described having to give up her job which had increased her social isolation.

Pamela *‘… I feel I’m all alone in the world. Struggling along with it. I gave up, I had a really nice job as a carer and I had to give that up because I was caring for my husband.’*

Similarly, another carer who looked after his mother continued to go to the café even after she passed away.

Walter: *‘… but the Saturday café is um, well you see what I want, my purpose now is just to meet people the same.’*

A daughter, caring for her mother also emphasised the challenges of trying to keep up paid work whilst supporting her mother and taking her out. In contrast to other carers, she emphasised that reason she goes to the café is for her mother’s benefit and she would not attend a café alone.

Moira *‘Not me, I wouldn’t, no. I don’t really feel the need, you know. I can imagine there are some carers who need time apart and probably need that, yes release and I think I’m aware of somebody maybe whose husband’s gone into a care home recently and so they still go, but no I wouldn’t do.’*

#### An opportunity for enjoying themselves and to switch off from being a carer in a safe environment

This theme incorporates what dementia cafés offer carers in a general, overall sense. Cafés are very positively regarded and carers look forward to and enjoy attending the cafés. Their overall enjoyment encompassed not only the activities provided and the company of others but also the feeling that the volunteers looked after them – something that carers often miss as they spend their time caring for others. The fact that the cafés are specifically for carers and people living with dementia meant they can relax in a safe environment.

Rachael: *‘… I get a lot of enjoyment, I do, I get a lot of laughter out of it, and um I find that I’m enjoying something, which is beneficial to my mum and to others, that’s what I get out of it*.’

Although some carers were unsure whether the person they supported was enjoying themselves, others were confident that they looked forward to and enjoyed attending the cafés. Knowing that the person living with dementia was enjoying themselves was very important for some carers.

Charlotte: ‘*My husband enjoys going, he looks forward to going, I mean when he gets there he can’t wait to get back out again but every time he’d looking forward to it, it’s um, sometimes he’s happy to go and sometimes not so much, but he does go so.’*

Importantly for some carers, cafés provide an opportunity for them and the person they supported to get out of the house, giving the day focus. It was something enjoyable that they could do together and was seen as an ‘occasion’.

Maria: ‘… *she likes the idea that we’ve gone somewhere together, like we’ve gone out together, like for her it’s like doing something with a friend and it’s different when someone just comes round for a cup of tea and you’re in your own home, there’s less of a, there’s less of an occasion.’*

Dementia cafés can offer relief from caring for carers by reducing the dependency of the person with dementia on the carer. Some of the cafés attended by the carer participants were organised so that carers met separately from the people with dementia. Carers could relax and be cared for themselves without having to worry about the person they cared for.

Diana: ‘*… she can go, quite often she’s on one table and I’m on another and it’s a safe environment.’*

Whilst there, little is expected of carers and those they care for in terms of how to behave and whether to join in with activities or not. Especially with the provision of food and drink, carers can also feel ‘looked after’ and supported by the volunteers even if they did not want to be sociable all the time.

Moira ‘*… you don’t have to do a thing, what do you want for tea, coffee and then there were sandwiches and cake and me, I suppose I didn’t appreciate how much I’d been doing until that happened because you just, you sit down and think, oh this is nice, so that was you know massive … really, really appreciated that.* … *you’ll sit down and relax and, you know, enjoy the fuss being made by the volunteers and the staff to give you your refreshments and so you just relax, I mean for me I would quite often not want to socialise actually because I’ll be exhausted and so it was in many ways fine with me.*’

Carers had mixed opinions about when attendees were most likely to gain the most benefit from going to cafés. Attending soon after diagnosis seemed to be important for some carers who said that immediately after the diagnosis they wanted to know more about dementia and by attending the café, they had gained a lot of useful information. However, several emphasised the value of continuing to attend even when the person with dementia had lost their speech and could no longer actively participate.

Maria: ‘*I guess the impact of that would have been greatest at the beginning, but actually at the time I didn’t realise that that would be the benefit, it was only later on I kind of thought to myself you know, it’s just been really useful for me to meet other people with dementia so that my knowledge of the condition is broader than just my mum.*’

Some carers emphasised that an important aspect of the café was feeling safe and having trust in the volunteers and coordinators. This meant they could relax and leave the person they cared for allowing them, for example, to go and spend time with other carers leaving their loved one with the café organisers and volunteers. Simply having other people there enhanced this.

Victoria: ‘*Yeah it’s safe and there are other people there. You know I’m there with him but now we, I mean we have to go everywhere together. I can never leave him in the house, you know, in the house on his own again.’*

#### Normalising living with dementia and being accepted

Importantly these cafés are a place where being a carer or having dementia are ‘normal’ and accepted. Many of the activities organised by the providers are unrelated to dementia - for example, chatting and singing are nothing to do with dementia and allow participants to simply to enjoy the moment. In this sense, cafés function as any other social club which brings people together to socialise and enjoy themselves but because everyone is in a similar situation, any unconventional or unusual behaviour can be more easily accommodated and tolerated. Similarly, openness about the challenges of being a carer of someone with dementia is acceptable.

Cafés are seen as safe places giving freedom for carers and the person with dementia to be themselves with others who understand them, in the knowledge that there is help at hand from volunteers if needed.

Diana: ‘… *my mother is a very sociable person, out of all of us in the family she’s the most sociable really and she’s the one who suffered the most from not going out and her friends didn’t seem to understand that… whereas in the memory cafés… and there are different people there and people who don’t mind if you’re saying the same things all the time.’*

According to some carers, cafés helped the person with dementia just be themselves.

Rachael: ‘*… when we go in there, they just have maybe sandwiches and a cake and cups of tea and juice and things, it’s not the quantity of what she has … she just, she’s just being herself, she gets to be herself.’*

Christopher: ‘*She enjoyed it… she could hardly dance because she had a stick but she loved dancing and singing… and the next minute she fell back and sat on someone’s knee… always laughing.’*

For one carer, it allowed her to learn about her mother from people who knew her long ago.

Rachael: ‘*I get to meet some of my, my mums old friends, you know so … she’s known them and they know, they know different parts of my mum, different, wherever they know my mum from, they will you know ‘Oh your mum is a lovely woman, I’ve known her for years’ or ‘I’ve known, do you know how long I’ve known your mum?’, it’s like, ‘Oh yeah, OK’, so out of it I get a lot of enjoyment, I do, I get a lot of laughter out of it, and I find that I’m enjoying something, which is beneficial to my mum and to others, that’s what I get out of it*.’

An almost inevitable aspect of attending cafés is comparison of yourself to other carers or comparison of the person they support with others with dementia. One carer highlighted how attending a café soon after her mother’s diagnosis was very useful because it helped her realise that people with dementia are actually quite ‘normal’ and not usually aggressive. However, this comparison could be a double edged because it also gave her a glimpse into what the future for her mother might hold as her dementia progressed.

Maria: ‘*… it’s just been really useful for me to meet other people with dementia so that my knowledge of the condition is broader than just my mum, you know. … but yes, I guess at the beginning it was like ‘Oh you know, people with dementia are normal people!’ You know, they just struggle with certain things, but they’re just normal nice people* … Y*es, but that does continue to be reinforced every time we go.’*

#### Peer support

This was a recurrent theme and was mentioned by all carer participants to a greater or lesser extent. Carers made frequent references to learning how others in similar situations had coped and felt able to ask for advice other carers. This aspect of the café is different to more general socialising in that other carers have a unique understanding of the difficulties of being a carer and can share their experiences of what can help them overcome these challenges.

Pamela: *‘You can actually discuss your situation with somebody else, who has got… in the same position as you are.’*

Some carers highlighted the benefits the person living with dementia could gain by being with other people with dementia.

Vera: *‘Even though he does not participate, he knows something is going on round and he could see other people … Ah, you know in the same category as him.’*

Maria: ‘… *it’s not, it’s not the activities particularly … she’ll take part in stuff, but it’s the sense of being with other people and getting involved in something rather than the particular thing that’s going on.*’

Importantly, when asked why they had initially attended a café, with the exception of one carer, most carers said because it had been suggested by a health care professional, rather than because they wanted to access peer support.

Charlotte: *‘I’ve got a feeling it might have been* (recommended by) *the nurses at the or it could have been the social worker. I’m not sure, somebody who was coming to see him at his flat mentioned it, and said about it, and said ‘Why don’t you try it?’ So we thought we’d try it and um, and that’s what we did, and we’ve been going ever since.’*

#### Developing social networks and reducing social isolation

Some carers said the reason they initially attended a café was to socialise, with one carer describing the café he attended as a social hub.

Walter: ‘*I mean it is important, well it’s more of a social hub really.*’

Attending the cafés sometimes led to the development of friendships. The fact that carers often met up with the same people each time often allowed these relationships to develop into friendships.

Pamela: *‘I’ve made quite a few friends and I’m quite good at remembering their names and one or two have asked for my phone number and I’ve asked for theirs and we do occasionally phone up, you know, I’ve said to them ‘Oh, come along.*’.

Importantly, some carers said that going to cafés helped reduce social isolation both for the carer and the person with dementia.

Perhaps because of their shared experiences as carers of people with dementia which provide an immediate bond, going to a café can provide a sense of belonging allowing carers to feel they are part of a group. Attending a café and being with other people helped them to feel connected with others.

Ranni: ‘(I enjoy) *… being in there, some nice food, talking to people and looking round and thinking who is doing what, and I become the part of the group, I’m not kind of carer sitting in the corner, I’m just become part of the whole thing, that is quite interesting*.’

One of the cafés had a number of male carers who regularly attended, and who over time, built up an informal peer support network.

Christopher: *‘Yeah, when I see George, that’s the other one there… and of course I, I pick another carer up and go to the dementia place on a Thursday, we go down to the Age UK for dinner and I give them a lift. I said I’m going there, you might as well get a lift … it’s nice to see him and have a little chat.’*

There was an acknowledgement of how as men, there were differences as well as similarities in their experiences

Christopher: ' …*different, like Peter down there, he goes on about his, his wife … he had all the help going and I never had that. … he used to go down other places because he got it good for him, you know, I don’t blame him, but, you know, he knew all the ins and outs of everything and he knew where to go and all this, but it’s good … I get to know him quite a bit more now because I see him quite a bit … I see another carer, he looks after two elderly couples, you know, one’s coming up to a hundred and the other ones in the mid-nineties … which I think is very good …’*

Interestingly, two of the participants were former carers and both were men who continued attending the café over a year after the person they cared for had passed away. The reason for this was primarily social.

Walter: [I’ve] *‘reached the end of the road with it and I’ve got to live my life now as happily as I can … to put it bluntly it’s just another little diversion uh in the form of socialising with people… I just do things now that please me, I like meeting people, talking to them, and um, it doesn’t mean the same as it did.’*

## Discussion

Our findings suggest that carers find these cafés to be welcoming, relaxed places to socialise and access support and information and echoing other research, the carers here also highlight the importance of peer support [20]. However, the depth of our analysis takes our understanding of the potential benefits for carers further. We have shown that despite diversity in carers’ needs and in how the cafés are organised, these cafés are enjoyable and provide a place where carers feel safe and able to be themselves with or without the person they care for. It is possible that the benefits to carers may go beyond what is offered by traditional carer support groups because of the many aspects provided by these cafés that carers find enjoyable and beneficial. Unlike many support groups, cafés simultaneously offer stimulating activities for both the person living with dementia and their carers, peer support and chances to socialise with others in a relaxed, café atmosphere. Carers can attend in the knowledge that any unusual behaviour will be accepted by other café attendees. Cafés can therefore reduce social isolation and provide some normality in their lives whilst simultaneously providing peer support and information. It is also possible that by referring these groups as ‘cafés’, it helps attending feel like something they might do with a friend or member of the family.

One very important finding here is how cafés may help to normalise dementia. They are a safe place where carers and people living with dementia can be themselves and can talk freely about any challenges they might be facing with café providers and other carers. Importantly cafés also give carers an opportunity to observe people living with dementia at varying points in the illness. This can help them feel more confident about the future and reassure them about any future challenges. Importantly, by attending the café, they learn about where they might access other types of support both from services and from other carers.

Carers’ descriptions of their experiences did not always suggest that the benefits of attending cafés were long-term but rather gave them something to look forward to and a focus for the day. However, some carers did say that cafés helped reduce social isolation and it was clear that cafés appear to facilitate the development of social networks and can be a source of information about dementia and support services suggesting a more lasting impact.

Another significant aspect of these cafés is that they are somewhere where carers can go alone or with the person they support. Some cafés effectively offer short-term breaks from caring or respite by allowing carers to go elsewhere. In other cases, volunteers entertained the people with dementia whist carers spent time with other carers in the building. For others though, cafés provided a space that encouraged them to interact and do activities with the person they supported [12].

The cafés also continued to be a welcoming place for two male carers over a year after the person they cared for had died. These examples suggest that dementia cafés have the potential to be seen as a ‘sustaining place’ [13] providing valued support even after the caring role has ended. This is particularly important as although the caring role can be difficult, the end of caring can require a long period of adjustment [25] and carers may benefit from the support of places such as dementia cafés.

In this manner cafés can help carers support their relationships. Depending on their circumstances and the point in their caring trajectory, carers differ in what they want and the flexibility in what cafés provide makes them an ideal form of support.

Our findings may be useful to practitioners setting up or running dementia cafés. Our carer participants have helped highlight how well these cafés are received and how much carers value them. A very important aspect of these cafés is the fact that carers feel welcomed and looked after. The activities are often appreciated but the significance of the relaxed, friendly atmosphere where carers and those they support feel that they can be themselves cannot be under-estimated.

### Study strengths

Our semi-structured method encouraged the participants to speak at length and to highlight aspects of experiences important to them. We interviewed carers of people with dementia who, particularly in the case of male carers, are not always heard. The participants were diverse and included carers in a variety of relationships with the person they cared for and who had been caring for differing lengths of time allowing us to conclude that they had many experiences in common with each other.

Furthermore, by using an open-ended approach and focussing on their perspectives, we were able to identify overarching aspects of their experiences such as feeling accepted and welcome at the cafés.

### Study limitations and future research

The limitations of this study need to be acknowledged. Firstly, we only interviewed carers who had attended cafés a minimum of three times in the last 6 months. This allowed us to learn about the experiences of more regular attenders but did not include those carers who no longer attended, for whatever reason. We therefore did not learn about the experiences of any carers who stopped attending because they or the person they cared for did not enjoy it. In the future, it would be valuable to interview carers who had stopped attending at various time points (for example, after only attending once or several times to explore their reasons for not attending).

The fact that few carers came from black and minority ethnic groups is also an acknowledged limitation. Future studies should ensure greater diversity in carer ethnicity given that evidence shows that the experiences of support services can be different for different ethnic groups [26].

We were only able to interview carers from cafés that gave us permission to do so. It is possible that this inadvertently meant that this self-selection reslted in us only speaking to carers from cafés whose organisers were sufficiently confident that carers would be positive about their experiences.

To give a more comprehensive picture and further our understanding of dementia cafés, future studies should also include people living with dementia. Furthermore, using mixed methods would allow the perceptions of a wider range of participants to be investigated. For example, the quantitative aspect would permit the measurement of any benefits using validated outcome scales whilst a qualitative approach would permit a more in-depth analysis of their experiences.

It would also be important to investigate the perceptions of café organisers and other service providers to explore what benefits for café attendees they are aiming to achieve. Clearer articulation of the intended benefits and outcomes would make it possible to determine if cafés are achieving their aims. This would potentially facilitate quantitative outcome measurement and although this might be difficult, might make an economic analysis possible.

It would be useful to monitor participant experiences over time. For example, a future study might interview attendees several times over e.g. a year. Using both qualitative and quantitative methods would provide useful information helping us understand how carers’ experiences change (or do not change) over time. This would facilitate clarification of   when cafés are most beneficial and how they might best be adapted to as dementia progresses.

Our study suggests that dementia cafés offer more to carers than more traditional support groups. Future studies might valuably compare the outcomes and experiences of those attending other support groups allowing this potential difference to be clarified and enhancing the support offered to carers of people with dementia.

None of the carers here were looking after someone with young onset dementia (YOD) - dementia diagnosed before the age of 65 years [27]. Although a relatively small group, the needs of those with YOD and their carers are thought to be especially challenging and isolating [28]. Given the value of these cafés in allowing carers to feel ‘normal’ and to gain peer support, such cafés may be particularly beneficial for them.

Finally, future research should investigate the potential of cafés for carers of people with a range of health conditions, not just those supporting someone with dementia.

## Conclusions

In conclusion, the diversity and preferences of people with dementia and their carers mean that it is challenging for café organisers to please all their attendees but we have demonstrated that whatever activities are offered, these cafés offer a unique type of support allowing carers and those they care for to be themselves without feeling embarrassed or stigmatised. Some carers benefit more from peer support and a relief from their caring responsibilities, whilst others enjoy the opportunities for joint activities with the person they care for in a non-judgemental, relaxed atmosphere.

## Additional file

Additional file 1: Interview Topic Guide. The topic guide was used to facilitate consistency between the interviewers and ensured that topics previously identified as important were covered. (DOCX 16 kb)
