# Supplementary material for: A qualitative study of carers’ experiences of dementia cafés: a place to feel supported and be yourself
Source: BMC Geriatr. 2017 Jul 25;17:164. doi: 10.1186/s12877-017-0559-4 (PMC5527402; doi:10.1186/s12877-017-0559-4)
Supplement: Additional file 1: — Interview Topic Guide. The topic guide was used to facilitate consistency between the interviewers and ensured that topics previously identified as important were covered. (DOCX 16 kb) [file 12877_2017_559_MOESM1_ESM.docx]

**Dementia café topic guide**

**Date:**

**Participant code:**

**Place of interview: (e.g. participants own home)**

**Participant has read and signed the consent form? (Y/N)**

**Has the participant completed the background information form? (Y/N)**

Before the interview starts, participants will be asked if they have any (further) questions about the study and also reminded of their right to withdraw from the study and that if they do not want to answer any specific questions, they don’t have to.

1. How do you usually get to the dementia café? Is it easy (or not) to get here?

2. How long have you been coming to the café?

3. Do you usually come with the person you care (name) for or do you tend to come alone?

4. Can you tell me why you first decided to come to the dementia café?

5. How have you found attending the café?

6. What do you enjoy most about attending? Is there anything you don’t enjoy or would like to change about the café?

7. *If the person with dementia also attends the dementia café:* Do you think the person you care for (name) enjoy coming to the dementia café?

8. Are there any benefits of downsides from coming to the cafes that last longer than just the time in the café?

9. Do you think that attending the dementia café has any benefits at particular times?

10. Are you receiving any other sort of support e.g. from the voluntary sector (probe any types of support)?

11. *If the carer is receiving other support*: How is the current support gained from the dementia café similar or different to other support?

12. Is there anything else you would like to say about your experiences of coming to dementia cafés?

**Thank you for helping us with our research!**
